# Are Body Composition Estimates Affected by the Menstrual Cycle in Females With or Without Hormonal Contraception?—A Case‐Control Study

**DOI:** 10.1002/ejsc.12283

**Published:** 2025-04-20

**Authors:** Daniela Stein‐Brüggemann, Laura Schultz, Katharina Malin Kiefer, Jan Fürst, Rüdiger Reer, Jan Schröder

**Affiliations:** ^1^ Medical School Hamburg Institute of Interdisciplinary Exercise Science and Sports Medicine Hamburg Germany; ^2^ University of Hamburg Faculty of Psychology and Movement Science Hamburg Germany

**Keywords:** air‐displacement plethysmography, bioelectrical impedance, body composition, caliper, eumenorrheic females, menstrual cycle, water retention

## Abstract

Hormonal‐induced water retention during the menstrual cycle (MC) may affect the estimates of body composition (BC) parameters depending on the MC phase if tissue hydration or volume is part of the BC analysis equations. Given this, MC phase‐dependent changes of BC parameters might be expected within females for bioelectrical impedance analysis (BIA) or air‐displacement plethysmography (ADP), whereas skin‐fold calipometry (CAL) might not be affected. This study aimed to evaluate BC analyses during a regular MC by means of BIA and ADP with CAL serving as a control method in females with or without hormonal contraception with males serving as a control group. In a case‐control design with repeated measurements, BC was determined using BIA, ADP, and CAL in 54 participants (age 18–33; BMI 17.0–27.8) divided into females using hormonal contraceptives (HC) (*n* = 19), females using no‐hormonal contraceptives (no‐HC) (*n* = 17), and males (*n* = 18). BC was assessed on four cycle‐related days (menstruation, late follicular, ovulation, and late luteal). There were only small intraindividual BC variations during the MC (CV% 0.5–5.2) and neither significant time effects within any group (*p* = 0.065–0.939) nor significant time*group interactions (*p* = 0.151–0.956) for all devices (BIA, ADP, CAL) in any BC parameter. The results indicate that hormonal‐induced water retention, if any, during MC had no effect on BC estimates of ADP, BIA, and CAL or were too small to be identified neither in females with HC nor in females with no‐HC.

**Summary**

- Despite physiological hormonally induced changes of body water content or retention, there were no significant alterations of total and extracellular water or body volume during the time course of a regular female menstrual cycle in eumenorrheic young females.
- Body composition assessments either in terms of bio‐electrical impedance analyses or by means of volumetric air‐displacement plethysmography showed only minor intraindividual variations during the menstrual cycle, not revealing any significant changes over time neither in fat mass nor in fat‐free mass parameters.
- These observations were not differing between females with or without hormonal contraception or males serving as controls.
- Body composition analyses showed to be independent of the female menstrual cycle leading to the assumption that women do not have to consider their menstrual phase when assessing their body fat or fat‐free mass in their athletic monitoring.

## 1 Introduction

Body composition (BC) assessment is crucial in clinical or high‐performance athletic environments to monitor either the lean body mass or body fat proportions (Kasper et al. 2021; Walowski et al. 2020). Except from cadaveric studies, body fat or lean body mass cannot be measured. Thus, body composition has to be estimated using various methodological approaches. In the environment of high‐performance athletes, there are some current methods being used and fulfilling practical and economic recommendations. The historically oldest approach, calipometry (CAL), showed to be most sensitive for diet or exercise‐related body fat percentage (BF%) changes. CAL estimates BF% from the sum of skinfold measurements. Theoretically, within a 2‐compartment model, it is possible to calculate additional parameters of body composition (BC), such as fat mass (FM) and fat‐free mass (FFM), using BF% and body weight (Kasper et al. 2021).

In the environment of the athletes monitoring, BC analyses have to be noninvasive as well as time and relatively cost‐economic, but each technical approach has its strengths and weaknesses—air‐displacement plethysmography (ADP) and bioelectrical impedance analysis (BIA) are widely spread commonly used methods for sports medical purposes (Kasper et al. 2021; Smale et al. 2013).

ADP calculates FM and FFM based on body weight and volume measures, enabling estimations of body density using Poisson's Law and the Siri equation considering the lung gas volume (Kasper et al. 2021). Air‐displacement leads to very similar BC results compared to the formerly accepted reference method, the water‐displacement (water weighing) with at least BF% overestimations of 1.3% and only small variability coefficients of about 1.7% (Kasper et al. 2021) indicating high reliability and external validity for BC assessment across various populations (Vasold et al. 2018).

BIA assesses the body's electrical properties by analyzing differences in conductivity and impedance between fat‐free mass (FFM) and fat mass (FM) (Marra et al. 2019). Eight electrode broad band multi‐frequency devices (bio‐electrical spectroscopy, BIS) are able to differentiate between extracellular and intracellular fluid by measuring the estimated resistance and reactance of various tissue types (Kasper et al. 2021; Kyle et al. 2004). However, the validity and reliability of BIA can vary significantly depending on the population studied and the specific devices used, which rely on distinct estimation equations (Bosy‐Westphal et al. 2013; Kasper et al. 2021; Talma et al. 2013).

As body volume is part of the ADP estimation equations and body water is part of the BIA estimates, both approaches might be affected by water retention and probable minor edema related to the menstrual cycle (MC), because elevated estrogen concentrations are accompanied by increases of extracellular fluid and elevated progesterone concentrations stimulate water retention, thereby increasing interstitial fluid (Carmichael et al. 2021; Stachenfeld and Taylor 2004). But to date, these assumptions are still a matter of debate. Some studies revealed MC‐related BC changes (Kanellakis et al. 2023; Tomazo‐Ravnik and Jakopič 2006), whereas others did not (Byrd and Thomas 1983; Cumberledge et al. 2018; Hicks et al. 2017; Koşar et al. 2022; Ong et al. 2022), maybe due to methodological variations, such as the chosen time points during MC and the use of hormonal contraceptives or not.

In a study by White et al. (2011), women recorded their symptoms daily in a journal over the course of 1 year, noting instances of bloating or fluid retention during their menstrual cycle. The results indicated that these sensations were most pronounced at the onset of menstruation. However, no significant correlations were found between estrogen and progesterone levels. This suggests that the clinical symptoms of fluid retention are not necessarily temporally linked to hormonal fluctuations.

The present study aimed to address this persistent research gap. We defined four time points of measurements according to the MC; first, directly after the start of menstruation characterized by the lowest estrogen concentrations, then the late follicular phase characterized by the highest estrogen concentrations peaking just before ovulation, followed by the day of ovulation and finally the late luteal phase showing increased estrogen and the highest progesterone concentrations (Blagrove et al. 2020; Schmalenberger et al. 2021; Thompson et al. 2021)—comparable to the latest approach of Kanellakis et al. (2023). With respect to probable suppression effects of endogenous hormones (Schaumberg et al. 2017), we distinguished the eumenorrheic younger female sample into those using hormonal contraception (HC) or not.

This study aimed to identify MC‐dependent changes of BC estimates, especially the BF% in terms of CAL, ADP, and BIA, and FM as well as FFM and their underlying measures like body volume or body water by means of ADP or BIA. As an extra topic, we searched for probable differences between women using HC or not.

We hypothesized that there were no effects within males and no effects for CAL measures at all, but we expected MC‐related changes within the female subsamples for ADP and BIA assessments due to MC‐related variations of the hydration state and subsequent body volume changes.

## 2 Methods

### 2.1 Study Design

The study was conducted as a two‐factorial case‐control design with repeated measurements (group*time). Participants were divided into three groups: females with no‐HC and—for comparisons—females with HC, as well as men serving as a control group (CG). All of them were examined at four occasions related to the female MC; males at the respective elapsed time points (t1: day 1–3 after start of menstruation, t2: day 10–11 after start of menstruation, t3: day of ovulation at about 14 days after start of menstruation, t4: day 20–21 after start of menstruation).

The precise day of ovulation was determined using a current regularly available drug store urine test kit (LH‐Strips‐sensitivity of 25mLU/mL; MomMed by Co‐ Innovation Biotech Co. Ltd; China).

### 2.2 Sample

An a priori power analysis (G*power V.3.1.9.7: Franz Faul, University of Kiel, Germany) resulted in a required sample size of *n* = 39 in total (within‐between effects for 3 groups × 4 time‐points: effect size *f* = 0.25, *α* = 0.05, power (1 − *β*) = 0.90) leading finally to a statistical power of 0.92. Assuming a drop‐out of 15%, at least 15 subjects per group were targeted.

The test subject had to be in good health, between 18 and 35 years of age, showing a BMI of less than 30 kg/m^2^. For inclusion, women had to have a regular eumenorrheic menstrual cycle. Per definition, HC was accepted only in the case of the pill or the ring. The menstrual cycle (MC) had to have a length of 28 days ± 2 days in the last 6 months. The categorization of the MC was carried out in collaboration with a gynecologist and based on clinical symptoms, which were recorded using a questionnaire.

The hormonal coil was excluded because the coil does not provide a hormonal pause like the other methods. Further exclusion criteria were conditions hampering the data assessment or affecting the body tissue hydration, for example, acute claustrophobia, metal implants, dieting or irregular menstrual cycles, high‐intensity training on the day of measurement, and injury, illness or any use of medication that could influence the results. This had to be clarified individually prior to testing.

**FIGURE 1:**
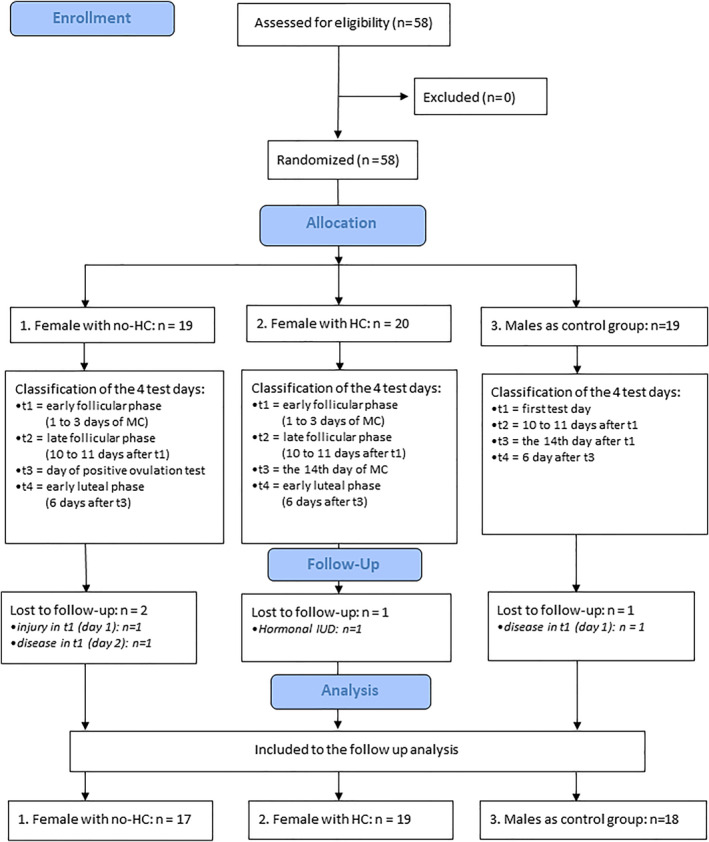
Flow chart of the study. HC = females using hormonal contraceptives; no‐HC, females using no‐hormonal contraceptives; MC = the menstrual cycle.

Subjects were recruited by word of mouth at universities and sport clubs and by posting flyers. It was determined that for a regular 28‐day MC (± 2 days), T3 (day of ovulation) corresponds to 14 days (± 2 days) after the start of menstruation. A positive ovulation test within this timeframe was always a prerequisite for the BC assessments; otherwise, the participant was excluded from the study. A total of 58 Caucasian volunteers were recruited and enrolled, of which only 4 dropped out during the observation period leading to a remaining sample of 54 subjects to be included into the statistical analyses: in detail, 19 males, 17 females (HC), and 18 females (no‐HC) (Figure 1).

Participants' age ranged from 18 to 33 years, and their BMI ranged from 17.0 to 27.8 kg/m^2^. Detailed sample characteristics are displayed in Table 1.

**TABLE 1:** Sample characteristics.

		Age (y)		Weight (kg)		Height (m)		BMI (kg/m^2^)	
	N	M	SD	M	SD	M	SD	M	SD
Females no‐HC	19	23.1	3.1	63.5	6.9	1.69	0.06	22.2	2.3
Females HC	17	24.8	4.4	68.8	10	1.72	0.08	23.3	2.5
Males	18	25.4	2.6	74.6^b^	6.5	1.82^a^	0.06	22.5	1.8
Total	54	24.4	3.6	68.9	9.1	1.74	0.09	22.6	2.3

^a^ Males different from females HC and no‐HC.

^b^ Males different from females no‐HC (post hoc Bonferroni testing after significant ANOVA).

All participants were informed about study goals and methods and the voluntary character of their participation, their right to withdraw as well as the privacy of the data management prior to the study and gave their written informed consent. The study was approved by the local ethic committee and was performed in accordance with the Declaration of Helsinki (World Medical Association 2013).

### 2.3 Body Composition Analysis

All participants were examined using three different measurement methods or technical approaches to estimate BC in a fixed order at approximately the same hour (± 1 h) of the respective day during the repeated measures covering a whole MC (Collins and McCarthy 2003). Each assessment at any time point was executed as a double measurement to make the data more robust against error confounders with the average of both measures being used as a dependent variable for the later statistical analyses. BC analyses always started with CAL, followed by ADP and finally BIA. All raters were experienced and familiar with the data acquisition procedures (Kasper et al. 2021). For standardization, participants were asked to empty the bowel and bladder and did not eat, smoke, or consume alcohol 2 hours prior to testing, or drink any fluids within 30 min prior to or during testing, respectively (Heyward and Wagner 2004).

#### 2.3.1 Calipometry (CAL)

For the present sample of normal weight to slightly overweight Caucasians, calipometry (CAL) was used as a simple noninvasive and inexpensive technical approach to determine BC from subcutaneous fat mass in terms of a 2‐component model (Kasper et al. 2021). Referring to the original Czech source of Jana Parizkova (1962), skinfolds at 10 sites of the body were measured leading to the sum of skinfolds (mm), which was then transformed into age and sex‐adjusted BF% values (based on specific regression equations) searched manually from database tables (Clasing and Siegfried 2002, 79–84). The respective were described earlier by Clasing and Siegfried (2002).

**FIGURE 2:**
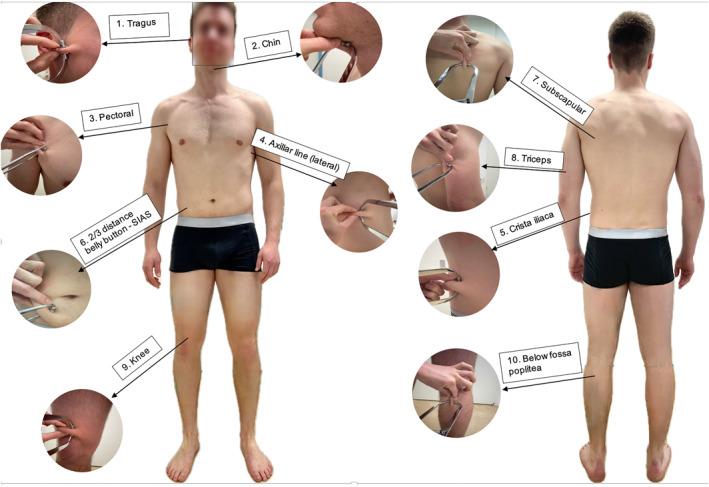
Calipometry (CAL), 10‐point skinfold thickness measurement referring to Parizkova (1962).

In summary, the method can be described as follows: The skin was pinched between the thumb and index finger and lifted 1–2 cm from the underlying tissues while maintaining the skinfold. A Harpenden Caliper (www.harpenden‐skinfold.com, pressure: 10 g/mm^2^, 0.2 mm resolution) was then used to measure the skinfold thickness twice, allowing the values ​​(in mm) to be averaged and summed from the head: tragus (1), beneath the chin (2), from the upper body front side: pectoralis axillar insertion (3), chest in the axillar line at the rip number 7 (4), directly above the iliac crest (5), the belly at the 1/3 distance between the belly button and superior anterior iliac spine (6), from the upper body back‐side: just beneath the scapulae triangle (7), at 50% of the length of triceps muscle belly at the upper arm backside (8), at the lower limb: upper edge of the knee cap at the thigh front side (9), and finally above the calve muscles at the back of the knee with slightly bent leg (10)—all sites from the same side of the body (Clasing and Siegfried 2002, 79) (Figure 2). BF% served as the CAL‐dependent variable for latter statistical analyses.

Methods such as the Parizkova 10‐point method, which sum skinfold thickness from various body locations, demonstrate strong agreement with whole‐body measurements using Dual energy x‐ray absorptiometry (DXA) (Ong et al. 2022). Skinfold thickness measurement is considered less influenced by daily activities, meal intake, and hydration status compared to other methods (Kasper et al. 2021).

The Harpenden's caliper as a special device is considered as the gold standard among skinfold forceps (Esparza‐Ros et al. 2019) demonstrating an excellent intrarater reliability (ICC: 0.944, 95%CI: 0.926–0.963) (Hoffmann et al. 2022). In our laboratory, we achieved even higher reliability coefficients from the double measurements at the first of four occasions (ICC_3.1_: 0.999, 95%CI: 0.998–0.999).

#### 2.3.2 Air‐displacement Plethysmography (ADP)

BC analyses based on ADP were performed using the BODPOD GS‐X (Cosmed, Rome, Italy)—the only ADP system using whole body densitometry. The technology is based on Poisson's law for calculating air displacement and thus for calculating volume. Density was calculated using measures of body mass and volume in order to calculate the body density after subtracting the thoracic gas volume from the total body volume. Isothermal air is then measured via inbuilt systems or generated via a prediction formula and combined to calculate a corrected body volume and body composition via numerous predictive equations (e.g., the Siri equations) to estimate FM, FFM, and BF%, representing a 2‐component model (Kasper et al. 2021).

The practical procedure may be described in brief according to Wagner et al. (2000).

Before each test, the BodPod was calibrated according to the manufacturer's instructions with the chamber empty using a cylinder of known volume (50 L). After assessing the body weight, the subject, in briefs and swim cap only, then entered and sat in the fiberglass chamber. The BodPod was sealed, and the subject breathed normally for 20 s while the body volume was measured.

Regularly, the thoracic gas volume may be determined by a relatively complex breathing maneuver: Subjects are connected to a breathing tube internal to the system to measure the thoracic gas volume (VTG). The subject resumed tidal breathing through the tube. After two or three breathing cycles, a valve in the circuit momentarily occluded the airway. At this point, the subject gently “puffed” by pressure fluctuations in the airway and chamber that were used to determine VTG. This value was used to correct the body volume for VTG (Wagner et al. 2000).

In our laboratory, the thoracic gas volume was determined using the manufacturer's estimation equations due to practical problems during the above‐mentioned maneuver needed for expiration volume measurements.

As a limitation, the method is sensitive to clothing, body hair, air movement, moisture, pressure, and temperature and moreover by its lack of ability to differentiate FM distribution (Higgins et al. 2001; Fields et al. 2004), but the device similar to the former gold standard for densitometry—water weighing—has shown to be valid and reliable (ICC: 0.996) (Mccrory et al. 1995; Muntean et al. 2023), and is noninvasive and time‐efficient due to the procedure's quick test time.

Body weight, body volume, BF%, FM, and FFM served as dependent ADP parameters for following BC statistical analyses. In our own laboratory, the test‐retest reliability calculated from the first of four double measurements demonstrated excellent coefficients for all variables (ICC_3.1_ > 0.99).

#### 2.3.3 Bioelectrical Impedance Analysis (BIA)

The medical body composition analyzer mBCA 515/514 (SECA, Hamburg, Germany) was used for BIA‐based BC analysis. The device offered four electrode pairs (both hands and feet in a standing position) to determine the tissues' impedance (R) and reactance (Xc) being processed to determine the tissues' water content, for example, total body water (TBW) and extracellular water (ECW), which in turn were used for the instrument‐specific regression equation to estimate FM, FFM, body water content, and subsequent BF%, meaning a 3‐component model of BC.

In comparison to other methods, BIA is relatively inexpensive and very time‐efficient. And using 4 electrode pairs (hands and feet, both body sides) makes the mBCA 515/514 a valid, reliable, and precise BC assessment device (Kasper et al. 2021). In our laboratory, the reliability of all the estimated BC parameters calculated from the first of four double measurements was excellent (ICC_3.1_ > 0.99).

As the waist circumference and the body height were part of the necessary information for the device‐specific regression formula, a tape measure and a stadiometer were needed as additional equipment for the BIA. FM, FFM, and BF%, as well as TBW and ECW served as BIA outcomes for statistical analyses.

### 2.4 Statistical Analysis

Data were described as mean (M) and standard deviation (SD). Normal distribution was confirmed using the Kolmogorov–Smirnov test. Intra‐individual variability within the four time points of measurements was calculated group‐wise as the standard error of the measurement (SEM = SD/√*n*) and coefficient of variation (CV% = SD/M*100) based on the individuals' means and standard deviations (t1–t4) being averaged afterward for the respective samples.

The t1 baseline values were analyzed in the context of a group comparison (men vs. women HC vs. women no‐HC) using a 1‐way ANOVA. A post hoc Bonferroni procedure was applied if the ANOVA indicated significance.

A 2‐way ANOVA with repeated measurements (general linear model) was conducted for significance testing of the interaction effect (time*group), with time being defined as MC‐related test points. For group‐wise analyses, a 1‐way ANOVA with repeated measurements (rANOVA) was applied. In the case of a violation of the sphericity assumption, the Greenhouse–Geisser correction was used to adjust *p*‐values. In the case of a significant time effect, multiple comparisons were corrected for cumulating *α*‐errors using the post hoc Bonferroni procedure. Significance was accepted at a *p*‐value ≤ 0.05.

All analyses were conducted using the R‐based statistical freeware JASP (V 0.18.1, University of Amsterdam, The Netherlands).

## 3 Results

The interaction effects did not reveal significance for any parameter and any device (*p* > 0.05, ranging from *p* = 0.151–0.956), which was only hypothesized for the CAL skin‐fold measures (*p* = 0.956), meaning that there were no sex‐depending differences between males and females of any BC parameters throughout the repeated measurements (MC) (Table 2).

**TABLE 2:** Body composition: descriptive statistics, variability, and ANOVA effects.

			t1		t2		t3		t4		Variability		1‐way rANOVA			2‐way ANOVA (time*group)		
			M	SD	M	SD	M	SD	M	SD	CV%	SEM	F‐value	*p*‐value	ŋ^2^p	F‐value	*p*‐value	ŋ^2^p
BF%	CAL	Males	14.6^a^	3.9	14.4	4.1	14.5	4.0	14.4	4.1	2.51	0.17	0.803	0.498	0.045	0.257	0.956	0.01
		Females HC	20.3	3.8	19.9	3.9	19.8	4.0	19.9	4.1	2.63	0.26	1.598	0.201	0.082			
		Females no‐HC	21.9	4.4	21.8	4.3	21.8	3.9	21.6	3.9	3.00	0.30	0.359	0.783	0.022			
	ADP	Males	14.6^a^	6.1	14.1	5.9	14.4	5.7	14.4	5.8	3.00	0.20	2.230	0.089	0.119	1.126	0.350	0.04
		Females HC	24.6	5.3	24.4	5.2	24.9	5.1	24.8	5.3	2.00	0.23	2.076	0.114	0.103			
		Females no‐HC	26.5	5.8	26.3	5.9	26.6	6.0	26.2	5.9	1.71	0.22	1.158	0.336	0.067			
	BIA	Males	14.7^a^	5.1	14.3	5.4	14.4	4.9	14.4	4.9	5.19	0.31	0.624	0.603	0.035	0.526	0.788	0.02
		Females HC	24.9	3.6	24.5	3.9	24.8	4.1	24.4	3.9	2.20	0.26	2.505	0.069	0.122			
		Females no‐HC	26.8	4.6	26.8	5.2	26.9	4.6	26.8	4.5	2.12	0.27	0.134	0.939	0.008			
Weight (kg)	ADP	Males	74.7^b^	6.7	74.5	6.4	74.9	6.7	74.9	6.9	0.69	0.26	1.721	0.174	0.092	0.696	0.653	0.03
		Females HC	63.5	7.1	63.3	7.1	63.4	7.0	63.5	7.2	0.58	0.19	0.813	0.492	0.043			
		Females no‐HC	68.8	10.3	68.4	9.9	68.7	10.1	68.6	10.0	0.75	0.26	1.191	0.323	0.069			
	BIA	Males	74.7^b^	6.7	74.5	6.4	74.9	6.7	74.9	7.0	0.66	0.25	1.369	0.263	0.075	0.691	0.657	0.03
		Females HC	63.5	7.1	63.3	7.0	63.3	7.0	63.5	7.2	0.58	0.19	0.835	0.481	0.044			
		Females no‐HC	68.8	10.3	68.4	9.9	68.7	10.1	68.6	9.9	0.74	0.26	1.215	0.315	0.071			
FM (kg)	ADP	Males	11.0^a^	5.0	10.6	4.8	10.9	4.7	10.9	4.7	3.82	0.17	2.564	0.065	0.131	0.781	0.586	0.03
		Females HC	15.7	4.4	15.6	4.3	15.9	4.3	15.9	4.4	2.62	0.19	2.060	0.116	0.103			
		Females no‐HC	18.4	5.5	18.2	5.4	18.5	5.6	18.2	5.5	2.26	0.19	1.503	0.226	0.086			
	BIA	Males	11.1^a^	4.3	10.8	4.4	10.9	4.1	10.9	4.0	5.11	0.23	0.705	0.553	0.040	0.351	0.909	0.01
		Females HC	16.0	3.7	15.7	3.8	15.9	4.0	15.6	3.8	2.23	0.17	2.516	0.068	0.123			
		Females no‐HC	18.7	5.2	18.6	5.5	18.8	5.3	18.6	5.2	2.27	0.20	0.275	0.843	0.017			
FFM (kg)	ADP	Males	63.7^a^	6.2	63.9	6.1	64.0	6.2	64.0	6.5	0.68	0.22	1.897	0.142	0.100	1.599	0.151	0.06
		Females HC	47.7	5.6	47.7	5.4	47.5	5.4	47.6	5.7	0.85	0.20	0.972	0.413	0.051			
		Females no‐HC	50.4	7.1	50.2	7.0	50.2	6.9	50.4	6.9	0.82	0.21	0.650	0.587	0.039			
	BIA	Males	63.6^a^	5.6	63.8	5.3	63.8	5.5	64.0	6.1	0.70	0.23	0.912	0.442	0.051	0.934	0.472	0.04
		Females HC	47.5	4.4	47.6	4.3	47.5	4.3	47.8	4.5	0.73	0.18	1.661	0.186	0.084			
		Females no‐HC	50.1	6.3	49.8	5.9	49.9	5.9	50.0	5.8	0.77	0.20	0.887	0.454	0.053			
TBW (L)	BIA	Males	46.3^a^	4.3	46.5	4.1	46.6	4.5	47.2	5.6	1.10	0.26	1.351	0.268	0.074	1.441	0.202	0.05
		Females HC	35.0	3.5	35.0	3.4	34.9	3.4	35.1	3.5	0.87	0.16	1.265	0.296	0.066			
		Females no‐HC	37.0	4.9	36.4	4.6	36.7	4.6	36.7	4.6	1.40	0.27	1.981	0.129	0.110			
ECW (L)	BIA	Males	18.2^a^	1.8	18.2	1.7	18.4	1.8	18.3	2.0	1.78	0.17	0.533	0.662	0.030	0.914	0.487	0.04
		Females HC	14.7	1.7	14.7	1.5	14.7	1.5	14.8	1.6	1.76	1.13	0.576	0.633	0.031			
		Females no‐HC	15.6	2.0	15.4	1.8	15.5	1.8	15.5	1.9	1.73	0.14	1.289	0.289	0.075			
Volume (L)	ADP	Males	70.1^b^	6.6	69.9	6.3	70.3	6.6	70.3	6.7	0.60	0.21	1.762	0.166	0.094	0.567	0.756	0.02
		Females HC	60.9	7.0	60.7	7.0	60.8	7.0	60.9	7.2	0.50	0.16	1.000	0.400	0.053			
		Females no‐HC	66.3	10.2	65.8	9.9	66.2	10.0	66.1	10.0	0.73	0.25	1.276	0.293	0.074			

Abbreiviation: ADP, air‐displacement plethysmography; BF%, body fat %; BIA, bioelectrical impedance analysis; CAL, calipometry; ECW, extra cellular water; FM, fat mass; FFM, fat‐free mass; HC, hormonal contraception; TBW, total body water.

^a^ Males different from females HC and no‐HC.

^b^ Males different from females HC (post hoc Bonferroni testing after significant ANOVA at baseline = t1).

Similarly, the 1‐way repeated measures ANOVA found no significant difference in any BC outcome between the four measurement time points within each group, separately (*p* > 0.05, ranging from *p* = 0.065–0.939), which was hypothesized for males but probably not for the female subsamples (Table 2). The intra‐individual variations (CV%) were ranging between 0.5% and 5.2% (Table 2).

In the group comparison, no differences were observed between the two groups of women. Only the men showed partial differences compared to both or only one of the women's groups (1‐way ANOVA and post hoc Bonferroni) (Table 2).

Summing‐up the results, the 2‐factorial analyses did not reveal any significant interaction effects in any of the applied BC assessment methods, neither in the common BC outcome variables (BF%, FM, FFM) nor in the underlying parameters hypothesized as being related to MC‐induced water retention (body weight, body volume, TBW, ECW) (Table 2).

Moreover, the group‐wise analyses also did not reveal significant time effects in any BC parameter assessed with any method (CAL, ADP, and BIA), not for males and not for one of the both female subsamples (Table 2).

## 4 Discussion

This study aimed to identify, if any, BC changes during a regular eumenorrheic female MC in females using hormonal contraception compared to females with hormonal contraception, which were not assumed for males. The hypothesized BC changes should be detectable in BC analyses based on ADP or BIA, but not in CAL. From a statistical point of view, a significant interaction effect between the groups (females _HC_, females _no‐HC_, and males) and time (t1–t4, meaning menstruation, late follicular, ovulation, and late luteal phase) would have indicated group‐specific BC changes during the MC—secondary group‐wise analyses would have indicated group‐specific BC alterations between the specific MC time points.

However, our findings were not in line with the recent findings of Kanellakis et al. (2023), Campa et al. (2022) or the earlier findings of Tomazo‐Ravnik and Jakopič (2006), who observed body weight and body water alterations during the MC. Kanellakis et al. (2023) reported body weight and corresponding ECW increases of approximately 0.5 kg during menstruation (meaning t1 in our design) compared to the first week after menstruation (meaning t2 in our design), and somehow similarly Campa et al. (2022) described body weight and TBW increases from ovulation to the early follicular phase and decreases back from early follicular to ovulation in elite female soccer athletes showing the highest values in the early follicular phase after menstruation due to fluid accumulation (meaning t1 in our design) and the lowest after ovulation (meaning t3 in our design), but Tomazo‐Ravnik and Jakopič (2006) reported the lowest values for body weight and TBW in the late follicular phase (meaning t2 in our design) and the highest values in the late luteal phase (meaning t4 in our design)—but this pattern was observed only in 65% of the participating younger women.

If it was the case that women demonstrated markedly high fluctuations (approximately 1.5–4.5 kg) in their body weight during the MC, those weight changes also led to corresponding significant differences in the water weighing estimated BF% values (Bunt et al. 1989). But in general, it has to emphasized that weight and water content fluctuations observed elsewhere did not lead to any accompanying significant MC‐induced BC alterations in terms of estimated BC outcomes such as FFM, FM, or BF%, respectively (Campa et al. 2022; Kanellakis et al. 2023; Tomazo‐Ravnik and Jakopič 2006). Kanellakis et al. (2023) investigated women in the age range 19–35 years described as reproductive age and conducted their BIA and CAL analyses twice a week during the complete MC—meaning eight occasions. Kanellakis et al. (2023) is one of the few studies that was able to demonstrate a cycle‐dependent effect on body composition. However, their significant findings may have been identified only because they did not perform an ANOVA followed by a post hoc test with a multiple comparison correction to determine whether there were differences between the baseline measurement and subsequent time points. Instead, all post hoc testing was conducted with the highest statistical power, which is not in line with standard practice. The significant results were likely obtained because the paired *t*‐test was inappropriately used, and an ANOVA would have been essential.

From a statistician's perspective, we hypothesized MC‐induced group‐specific BC changes, but statistical testing did not reveal significance. But it has to be pointed out that not rejecting the null hypothesis is not equal to the fact that there is no effect. Probably and in line with Byrd and Thomas (1983), we should admit that systematic alterations were just not large enough to be statistically significant, which may be explained by our observed small BC variations of 0.5%–5.2% during the four occasions of BC analyses (Table 2).

Ong et al. (2022) investigated BC during the MC using DXA, ultrasound, and skinfold measurements in women with a eumenorrheic cycle. The women were measured at four different time points: early follicular phase, mid to late follicular phase, mid luteal phase, and second early follicular phase. Similarly, no significant changes in body composition were observed across the different phases of the cycle or among the various measurement methods.

Thus far, our results seem to be in a row with other studies not revealing significant MC‐related fluctuations of body water content, body volume, or estimated BC parameters like FM, FFM, or BF% in terms of BIA (Cumberledge et al. 2018; Hicks et al. 2017) and either ADP and Dual‐energy‐X‐ray‐absorptiometry concluding that BC may be assessed irrespective of the MC phase and regardless of hormonal contraception, despite feelings of bloating (Hicks et al. 2017; Ong et al. 2022). Koşar et al. (2022) investigated recreationally active young women during the mid‐follicular and mid‐luteal phases using DXA and BIA. Similar to other studies, no significant changes were observed between the different phases of the MC. The high variability was the sole reason for recommending repeated measurements of BC during the same phase of the MC.

Probably, the investigated population, the BC analysis devices with their technical specifications and equations and the chosen number of assessments during the MC as well as the use of hormonal contraception and the variety of hormone preparations and its distribution among females might be confounding influences being able to explain the inconsistent study results and their conclusions (Cumberledge et al. 2018; Kanellakis et al. 2023).

Given that the hormone concentrations change during the eumenorrheic MC with peaking estrogen concentrations in the late follicular phase accompanied by increased extracellular fluid and highest progesterone concentrations in the late luteal phase characterized by water retention in the interstitial fluid (Carmichael et al. 2021; Stachenfeld and Taylor 2004), it is obvious that the number and specific time of BC assessments is important to identify probable hormone‐induced water retention effects on the body water content and body volume. Hormonal contraceptives, like oral contraceptives, can modify these hormonal effects by suppressing endogenous hormone production (Schaumberg et al. 2017).

Kanellakis et al. (2023) investigated women in the age range 19–35 years described as reproductive age and conducted their BIA and CAL analyses twice a week during the complete MC—meaning eight occasions. Cumberledge et al. (2018) used four time points—comparable to our design. Tomazo‐Ravnik and Jakopič (2006) examined their sample of younger women at only three occasions during the MC. Campa et al. (2022) as well as Hicks et al. (2017) chose only two occasions (ovulation and early follicular after menstruation) ignoring the late luteal phase with high progesterone concentrations and associated water retention effects.

On the other hand, Hicks et al. (2017) investigated women with natural cycles or using hormonal contraceptives (HC) in order to distinguish between these subsamples considering probable HC suppressing effects on endogenous hormone concentrations, which was not considered in many studies. Byrd and Thomas (1983) found that a body weight change of 0.5 kg is not substantial enough to impact body composition. It can be assumed that the sensitivity of the devices is not high enough to detect such a minor change in the parameters. However, there is still a lack of consensus on the definition of MC phases and phase durations, as well as on the establishment of hormone profiles to determine precise measurement points (Elliott‐Sale et al. 2021).

### 4.1 Strengths and Limitations

It might have limited our study results that we observed only 1 MC. Moreover, we did not monitor the dietary habits probably affecting the BC, and we had no access to blood sampling facilities in order to monitor the actual state of female sexual hormone concentrations. But the excellent reliability coefficients support that we achieved a high standardization concerning data assessment conditions. Probably, we chose a needed power of 90% and a moderate effect size that were not suitable to identify questionable effects, but our design involved double measurements to eliminate random errors covering probably the assumed hormone‐induced water retention effects and we implemented a male control group as well as a control assessment tool (CAL) to strengthen possible observations in the primarily interesting female subsample using no HC leading to reasonable large subsamples for the monitoring of four time points covering a complete MC. Another limitation is that participants did not keep a daily diary to record their cycle‐related symptoms. Additionally, body composition was assessed in women who did not explicitly report experiencing water retention. Consequently, no group‐level statistical significance could be established.

## 5 Conclusion

Our data suggest that MC‐related physiological effects on water retention and subsequently assumed effects on body water content (BIA) or body volume (ADP) do not affect BC analysis results irrespective of the phase of the female MC, neither in females with or without HC. Assumed systematic variations, if any, remained beneath a detectable threshold.

Given this, it may be concluded that BC analyses in terms of CAL, ADP, and BIA are not susceptible to being hampered or confounded by the time point of measurement throughout the MC.

Future research might probably target to evaluate further specific populations, for example, other BMI clusters or age groups and perhaps observation periods covering more than 1 MC and a daily diary to strengthen the present study results. It would also be helpful to establish a consensus through an expert panel regarding the definition of MC phases, phase durations, and the development of a standardized hormone profile.

## Ethics Statement

The study was approved by the local ethic committee (number: MSH‐2023/272) and was performed in accordance with the Declaration of Helsinki.

## Consent

All participants were informed prior to the study and gave written informed consent was obtained from all subjects. All data relevant to the study were stored pseudonymously. After 10 years (no later than July 15, 2033), this data will be deleted.

## Conflicts of Interest

The authors declare no conflicts of interest.
